# Cavotricuspid isthmus ablation for atrial flutter guided by contact force related parameters: A systematic review and meta-analysis

**DOI:** 10.3389/fcvm.2022.1060542

**Published:** 2023-01-06

**Authors:** Naidong Pang, Jia Gao, Nan Zhang, Min Guo, Rui Wang

**Affiliations:** ^1^Department of Cardiology, First Hospital of Shanxi Medical University, Taiyuan, Shanxi, China; ^2^The First Clinical Medical College, Shanxi Medical University, Taiyuan, Shanxi, China

**Keywords:** atrial flutter, cavotricuspid isthmus, catheter ablation, contact force, ablation index

## Abstract

**Background:**

Contact force (CF) and related parameters have been evaluated as an effective guide mark for pulmonary vein isolation, yet not for linear ablation of the cavotricuspid isthmus (CTI) dependent atrial flutter (AFL). We thus studied the efficacy and safety of CF related parameter-guided ablation for CTI-AFL.

**Methods:**

Systematic search was performed on databases involving PubMed, EMbase, Cochrane Library and Web of Science (through June 2022). Original articles comparing CF related parameter-guided ablation and conventional parameter-guided ablation for CTI-AFL were included. One-by-one elimination, subgroup analysis and meta-regression were used for heterogeneity test between studies.

**Results:**

Ten studies reporting on 761 patients were identified after screening with inclusion and exclusion criteria. Radiofrequency (RF) duration was significantly shorter in CF related parameter-guided group (*p* = 0.01), while procedural time (*p* = 0.13) and fluoroscopy time (*p* = 0.07) were no significant difference between two groups. CF related parameter-guided group had less RF lesions (*p* = 0.0003) and greater CF of catheter-tissue (*p* = 0.0002). Touch-up needed after first ablation line was less in CF related parameter-guided group (*p* = 0.004). In addition, there were no statistical significance between two groups on acute conduction recovery rates (*p* = 0.25), recurrence rates (*p* = 0.92), and complication rates (*p* = 0.80). Meta-regression analysis revealed no specific covariate as an influencing factor for above results (*p* > 0.10).

**Conclusion:**

CF related parameters guidance improves the efficiency of CTI ablation, with the better catheter-tissue contact, the lower RF duration and the comparable safety as compared with conventional method, but does not improve the acute success rate and long-term outcome.

## 1. Introduction

Cavotricuspid isthmus (CTI) dependent atrial flutter (AFL), also known as typical AFL, is the most common macro-reentrant atrial tachycardia which can lead to thromboembolic events and heart failure (1). As the critical zone of slow conduction, CTI is the ideal target to interrupt the reentrant circuit (2, 3). Currently, radiofrequency (RF) catheter ablation (CA) has become an effective treatment for CTI-AFL (4, 5), whereas there is still a recurrence rate of more than 5% which often related to re-connection of isthmus (6).

Previous studies have shown the importance of transmural lesion for effective and long-lasting RF lesion (7). Sufficient contact between the ablation catheter tip and the target tissue is crucial to achieve transmural necrosis and scar formation (8), whereas excessive tissue contact may be potentially hazardous (9). Therefore, optimal tissue contact has become one of the pursuits of electrophysiologists for RF ablation. Although traditional CTI ablation can indirectly observe catheter-tissues contact using a combination of qualitative measures, such as reduced catheter movements and electrogram amplitudes, these methods are not accurate enough. With the development of ablation-assisted techniques, more parameters have been available for quantitative assessment of catheter-target tissue contact. Contact force (CF) as a new parameter reflecting the real-time catheter tip-tissue contact, had been confirmed to help transmural lesion formation in pulmonary vein isolation (PVI) (10). Previous studies have suggested that use of optimal CF value during PVI is associated with increased lesion volumes, and provides greater security in complication risks such as cardiac perforation (11, 12). In addition, in recent years, there are some CF related parameters, which have similar principle and function with CF, and also reflect the catheter-tissue contact, have been utilized in AFL ablation. Ablation index (AI) is an updated parameter of atrial fibrillation (AF) ablation, which incorporates CF, time, and power though a special algorithm, and can be reflected in real time during the ablation (13). Electrical coupling index (ECI) is another catheter-tissue contact related parameter created for AF ablation. ECI does not give direct information about the pressure of catheter tip-tissue interface, but reflects the real-time complex impedance and describes characteristic of tissue heating and lesion formation.

The effect of these parameters in achieving transmural lesion has been demonstrated in AF ablation (14–16), but there is a lack of evidence that they are equally effective in CTI-AFL ablation. Recently, there have been some relevant studies on influences of CF related parameters in CTI-AFL ablation, but results of these small, single center studies were not completely same. Accordingly, we conducted a meta-analysis to assess feasibility, safety and efficacy of CTI-AFL ablation guided by CF related parameters.

## 2. Methods

### 2.1. Data sources and search strategies

This meta-analysis was performed referring to established methods (17). An electronic databases search was performed on PubMed, EMbase, Cochrane Library and Web of Science (from inception to July 2022) by two independent reviewers (N.P., J.G.), using the following search terms: “atrial flutter,” “cavotricuspid isthmus,” “catheter ablation,” “radiofrequency ablation,” “contact force,” “ablation index” and “electrical coupling index” with no language restriction. Additional literature was further searched from review articles and references of relevant researches manually. Any discrepancies were arbitrated by the third reviewer (R.W.).

### 2.2. Study selection and quality assessment

Inclusion criteria were applied as follows: (a) randomized-controlled trials (RCTs) and observational studies on RF ablation of CTI-AFL; (b) compared to procedural parameters and clinical outcomes between CF related parameters-guided and conventional parameters-guided ablation; (c) baseline information and outcome data were complete and accurate. Reviews, case reports, editorials, single cohort studies, and animal studies were excluded. Included studies were not restricted by race, sex, age, or research country.

Two independent reviewers (N.P., J.G.) formally performed quality assessment. RCTs were evaluated using the Cochrane Collaboration bias risk assessment tool (18). Non-RCTs were assessed using the Newcastle-Ottawa Scale (NOS), with scores varying from 0 to 9 depending on the quality of studies, and papers were considered high quality if they scored 7 or higher. Any disagreements were adjudicated by the third reviewer (R.W.).

### 2.3. Data extraction

Two reviewers (NP and JG) independently extracted the data from the original articles and raw data files of all eligible studies, and entered into a predetermined spreadsheet as follows: (a) study information (first author’s name, published year, research country, type of study design, sample size, parameters that guide ablation); (b) participant characteristics (mean age, male gender, race, and baseline characteristics); (c) outcome indicators: RF duration, total procedural time, fluoroscopy time, RF lesion numbers, average CF values, acute conduction recovery, touch-up needed, recurrence/re-conduction rates, and risk of complications.

### 2.4. Statistical analysis

Statistical analyses in this meta-analysis were performed with Review Manager (RevMan, version 5.3) and Stata (version 12.0). The mean difference or standard mean difference and respective 95% confidence intervals (95% CI) were used as the measure of data for continuous variables, risk ratio (RR) and respective 95% CI were used as the measure of data for binary variables. If the heterogeneity across studies was less than 50%, data were pooled using fixed-effect model, otherwise, the random-effect model was used. Statistical significance was set as *p*-value of less than 0.05. Data of continuous variables represented by median and interquartile range were converted to mean and standard deviation to perform data synthesis and statistical analyses after checking for the normal distribution (19, 20). Heterogeneity was assessed by calculating *I*^2^ and Cochran Q test, with *I*^2^ value more than 50% or *p*-value of the Q test less than 0.1 was considered evidence of significant inconsistency (21, 22). If heterogeneity was present, sensitivity analysis was conducted to inspect the effect of a single study on the overall risk estimate by omitting one study at a time. Meta-regression analysis was also performed to examine the sources of differences among studies. If a particular covariate had a significant effect on heterogeneity (*p* < 0.10), further subgroup analysis was performed. We generated funnel plot to assess potential publication bias, and the asymmetry of the plot was evaluated by Egger’s test, with *p*-value of less than 0.05 indicating apparent asymmetry.

## 3. Results

### 3.1. Search results

**FIGURE 1:**
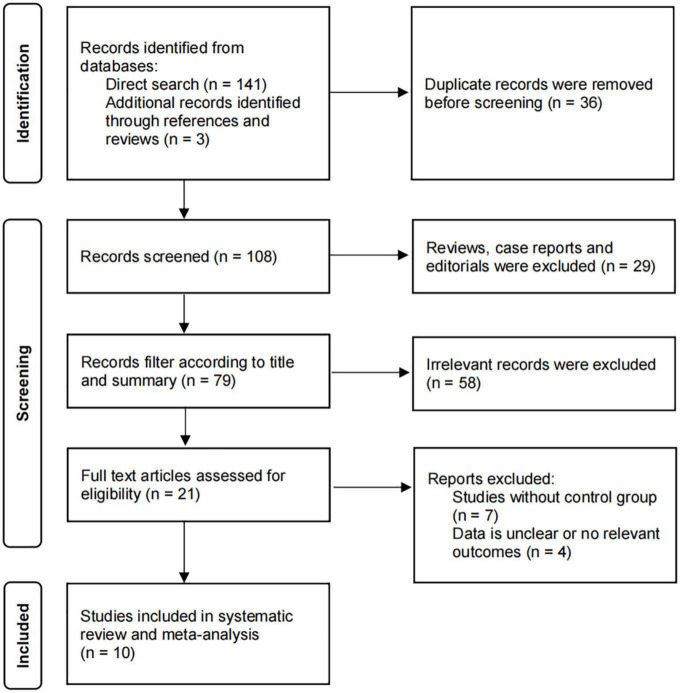
Flow diagram for study identification and inclusion.

The thorough literature search resulted in 144 records (141 from electronic databases and three from manual search). 36 duplicate studies were removed from the search results. After screening based on the inclusion and exclusion criteria, 21 studies were selected for full-text review. Of these, 11 studies were further excluded because no clear outcome data or no control group were available. Ten studies were eventually identified and included in the meta-analysis, which involved four RCTs (23–25) and six observational studies (26–31). Figure 1 shows the flowchart of the inclusions and exclusions.

### 3.2. Study characteristics and quality assessment

Included studies were conducted in centers across the United Kingdom, Canada, Denmark, Australia, and Japan. A total of 761 patients were enrolled in the entire cohort, 437 (57.4%) underwent ablation guided by CF related parameters and 324 (42.6%) underwent conventional parameters-guided ablation (control group). The mean age of the participants was 66.1 years, and there were predominantly males (78.2%). Regarding the ablation parameters, seven studies performed procedures guided by CF parameter, two guided by ECI, and one guided by AI. The summary of study characteristics is presented in Table 1. All included studies performed procedures using point-by-point ablation from the tricuspid valve annulus to the inferior cava veins, and clearly verified the bidirectional block after ablation and at procedure end by a separate double potential at the distal bipoles of an ablation catheter during the CS pacing and differential pacing maneuvers.

**TABLE 1:** Summary of included studies.

References	Research country	Study design	Parameter	Sample size	Age mean (±*SD*)	Males *n* (%)
Begg et al. (23)	UK	RCT	CF	53	64.0 (11.5)	45 (84.9)
Begg et al. (23)	UK	RCT	ECI	45	63.7 (10.9)	39 (86.7)
Boles et al. (26)	Canada	Retrospective study	CF	38	67.4 (9.5)	26 (68.4)
Giehm-Reese et al. (24)	Denmark	RCT	CF	156	67.6 (9.7)	120 (76.9)
Gould et al. (27)	Australia	Retrospective study	CF	60	64.0 (9.5)	47 (78.3)
Gül et al. (28)	Canada	Retrospective study	CF	37	66.2 (10.0)	26 (70.3)
Jones et al. (25)	UK	RCT	ECI	101	65.5 (11.0)	79 (78.2)
Sakama et al. (29)	Japan	Prospective study	AI	181	68.2 (9.7)	133 (73.5)
Saraf et al. (30)	UK	Retrospective study	CF	20	65.5 (8.5)	19 (95.0)
Venier et al. (31)	Canada	Retrospective study	CF	70	62.7 (10.9)	61 (87.1)

UK, United Kingdom; RCT, randomized controlled trial; CF, contact force; ECI, electrical coupling index; AI, ablation index.

**FIGURE 2:**
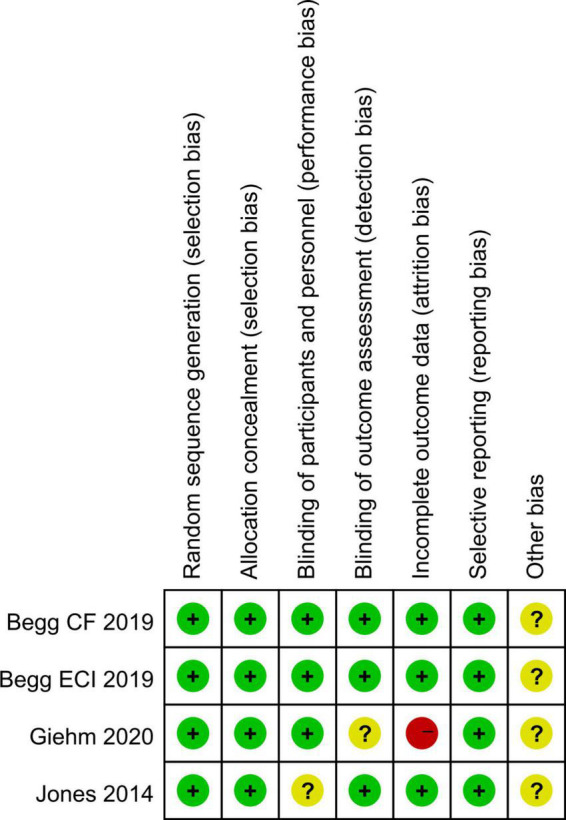
Risk of bias summary of included RCTs in the meta-analysis.

The four RCTs were assessed as high quality according to the Cochrane Collaboration criteria, although partial possible biases were unclear. The six observational studies were classified as high quality based on at least seven point of NOS scores, indicating a low risk of bias and suitable for analysis. Figure 2 and Table 2 show the results of quality assessment for RCTs and observational studies, respectively.

**TABLE 2:** Quality assessment of non-RCTs.

References	Study design	NOS score
Boles et al. (26)	Retrospective study	8
Gould et al. (27)	Retrospective study	7
Gül et al. (28)	Retrospective study	7
Sakama et al. (29)	Prospective study	7
Saraf et al. (30)	Retrospective study	9
Venier et al. (31)	Retrospective study	8

RCT, randomized controlled trials; NOS, Newcastle-Ottawa Scale.

### 3.3. Outcomes of procedural parameters

The differences of RF duration, procedural time, fluoroscopy time, average CF values, and number of RF lesions were assessed between the two groups.

**FIGURE 3:**
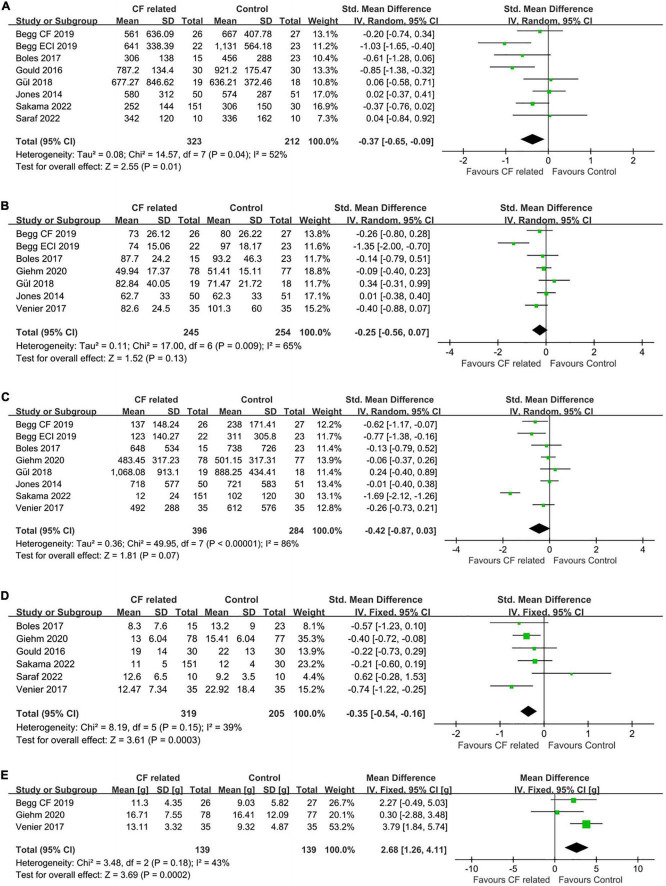
Forest plots comparing **(A)** RF duration, **(B)** total procedural time, **(C)** fluoroscopy time, **(D)** number of RF lesions and **(E)** average CF values between the CF related parameter-guided group and the conventional parameter-guided group.

RF duration was available in eight studies. Of these, five studies favored the guided by CF related parameters, whereas three studies had the opposite results. Pooled analysis showed that CF related parameters-guided ablation had a statistically significant shorter RF duration (SMD: –0.37, random-effect model, –0.65 to –0.09, *p* = 0.01; Figure 3A) with moderate heterogeneity across studies (*I*^2^ = 52%, *p* = 0.04 of *Q*-test).

In addition, there were seven studies and eight studies, respectively, reported the procedural time and fluoroscopy time. The results of quantitative synthesis showed that CF related parameters-guided ablation was associated with shorter procedural time (SMD: –0.25, random-effect model, –0.56 to 0.07, *p* = 0.13; moderate heterogeneity, *I*^2^ = 65%) (Figure 3B) and fluoroscopy time (SMD: –0.42, random-effect model, –0.87 to 0.03, *p* = 0.07; high heterogeneity, *I*^2^ = 86%) (Figure 3C) compared to conventional parameters-guided ablation, but did not reach statistical significance.

Regarding the number of RF lesions to achieve CTI bidirectional block, analysis of available data from six studies showed that ablation guided by CF related parameters was associated with less RF lesions compared to that guided by conventional parameters (SMD: –0.35, fixed-effect model, –0.54 to –0.16, *p* = 0.0003; low heterogeneity, *I*^2^ = 39%) (Figure 3D).

Average CF values during ablation were reported in five of the seven studies on CF parameter, with data from three studies of which were available for comparison. The average CF values of CF-guided group was 15.1 grams (g) (from five studies), while that was 13.2 g of control group. Pooled analysis showed that CF-guided ablation was characterize by significantly increased catheter-tissue contact (MD: 2.68, fixed-effect model, 1.26–4.11, *p* = 0.0002; Figure 3E) with low heterogeneity (*I*^2^ = 43%, *p* = 0.18).

### 3.4. Ablation efficacy and safety

**FIGURE 4:**
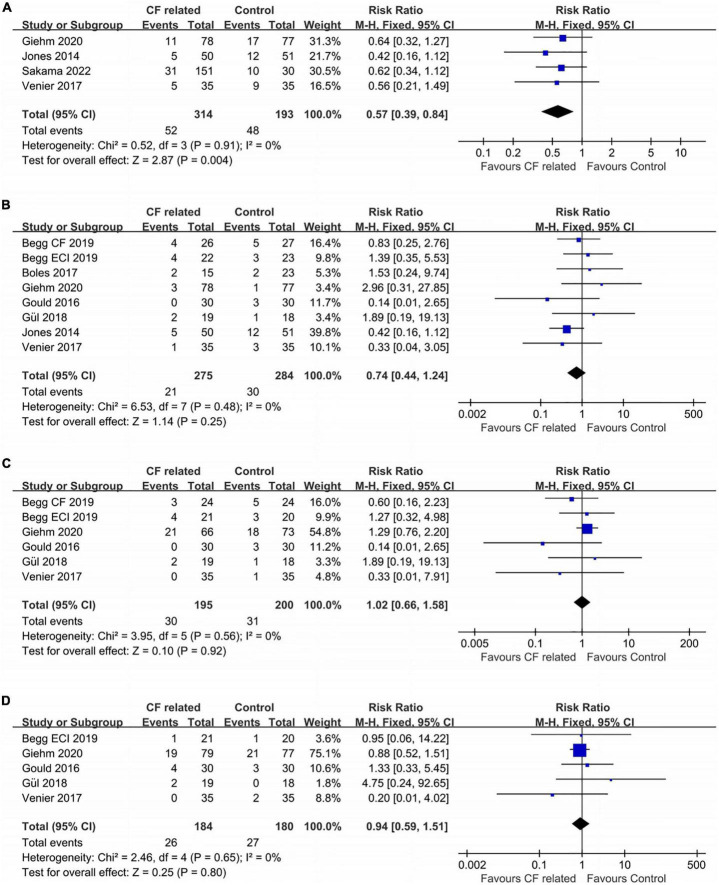
Forest plots comparing the **(A)** touch-up needed after first ablation line, **(B)** acute conduction recovery, **(C)** recurrence risk and **(D)** ablation complications between the CF related parameter-guided group and the conventional parameter-guided group.

Ablation guided by CF related parameters was associated with a higher success rate of bidirectional isthmus block after first ablation line achieved according our analysis, which meant less cases needed touch-up (16.6% vs. 24.9%, RR: 0.57, fixed-effect model, 0.39–0.84, *p* = 0.004; Figure 4A). An extremely low heterogeneity was observed (*I*^2^ = 0%, *p* = 0.91).

A total seven studies reported the information of acute conduction recovery after procedures (approximately 20–30 min after achieving CTI bidirectional block). There was no significant difference between the two groups (7.6% vs. 10.6%, RR: 0.74, fixed-effect model, 0.44–1.24, *p* = 0.25; Figure 4B) with an extremely low heterogeneity across studies (*I*^2^ = 0%, *p* = 0.48).

In terms of recurrence rates, ablation guided by CF related parameters showed similar risk as the control group (15.4% vs. 15.5%, RR: 1.02, fixed-effect model, 0.66–1.58, *p* = 0.92; Figure 4C). *I*^2^ was 0%, which indicated an extremely low heterogeneity.

The ablation complication risks of the two groups were no statistically significant which the data were obtained from five studies (RR: 0.94, fixed-effect model, 95% CI, 0.59–1.51, *p* = 0.80; Figure 4D). An extremely low heterogeneity was found (*I*^2^ = 0%, *p* = 0.65).

### 3.5. Publication bias assessment for included studies

**FIGURE 5:**
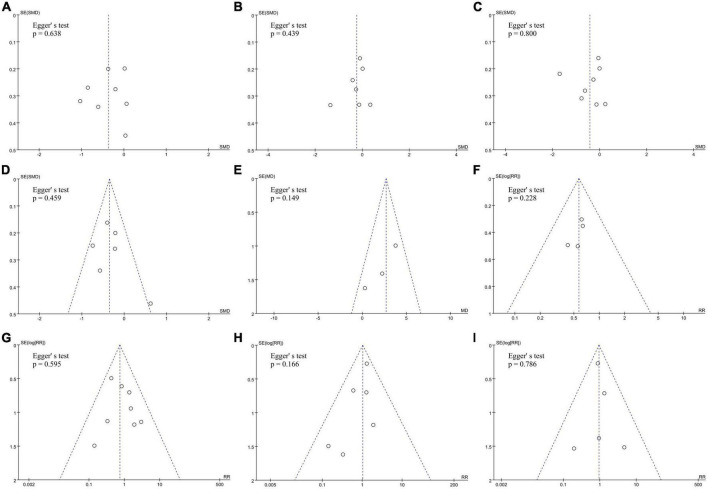
Assess the publication bias of **(A)** RF duration, **(B)** total procedural time, **(C)** fluoroscopy time, **(D)** number of RF lesions, **(E)** average CF values, **(F)** touch-up needed after first ablation line, **(G)** acute conduction recovery, **(H)** recurrence risk and **(I)** ablation complications by funnel plots and Egger’s tests.

No significant publication biases were found in all nine observed indicators of included studies by funnel plots and Egger’s tests (Figure 5).

### 3.6. Sensitivity analysis and subgroup analysis

Sensitivity analysis was performed to examine the solidity of the results of this work and the sources of heterogeneity between studies. The results of omitting one study at a time showed no significant change in research results and heterogeneity, except for one indicator (the same as in the subgroup analysis).

The intervention group of this study included a total three parameters (CF, ECI, and AI), we thus further performed subgroup analysis to separately evaluate the impact of each parameter on CTI ablation. Since the comparison of average CF values only contained CF parameter, so it was not included in the subgroup analysis. The results of the other six outcome indicators showed that only pooled analysis on fluoroscopy time had significant inter-group heterogeneity (*I*^2^ = 94.6%). The source of heterogeneity was the study in AI subgroup, which supported a significant reduction in fluoroscopy time in the intervention group, while the remaining subgroups showed no significant difference (Supplementary Figure 1). Heterogeneity between subgroups of the other outcome indicators was extremely low (*I*^2^ = 0%), indicating that different intervention parameters have little influence on the results.

### 3.7. Meta-regression analysis

The source of heterogeneity was further explored through meta-regression analysis. Covariates included published year, country where research was conducted, study design (RCT or observational study), interventional parameters (CF, ECI, or AI), mean age and male proportion of participants. The detailed results of the meta-regression analysis are showed in Table 3. All of above included covariates are not the main factors affecting heterogeneity between studies (*p* > 0.10).

**TABLE 3:** Results of meta-regression analysis for outcome indicators.

Variable	Slope coefficient	Standard error	*Z*-value	*P*-value	95% CI	
					**Lower limit**	**Upper limit**
**RF duration**						
Published year	-0.041778	0.1990302	-0.21	0.868	-2.570697	2.487141
Research country	0.0478591	0.8627267	0.06	0.965	-10.91412	11.00984
Study design	-0.4796924	1.918133	-0.25	0.844	-24.85189	23.8925
Interventional parameter	-0.3700575	1.088199	-0.34	0.791	-14.19694	13.45682
Mean age	0.352579	0.5520906	0.64	0.638	-6.662397	7.367555
Male gender	3.60509	9.821638	0.37	0.776	-121.1907	128.4008
**Procedural time**						
Published year	-0.2000909	0.1660483	-1.21	0.441	-2.309935	1.909753
Research country	-0.0383034	0.515673	-0.07	0.953	-6.59055	6.513943
Study design	-0.2750035	0.6548081	-0.42	0.747	-8.595129	8.045123
Interventional parameter	-0.9803946	0.8344757	-1.17	0.449	-11.58341	9.622624
Mean age	0.1243999	0.1646027	0.76	0.588	-1.967075	2.215875
**Fluoroscopy time**						
Published year	-0.1397646	0.0628184	-2.22	0.269	-0.9379486	0.6584194
Research country	0.9589619	0.59878	1.6	0.355	-6.64926	8.567184
Study design	-1.693153	0.9998991	-1.69	0.340	-14.39808	11.01177
Interventional parameter	-0.4049631	0.1679673	-2.41	0.250	-2.53919	1.729263
Mean age	-0.8643761	0.6181539	-1.4	0.395	-8.718766	6.990013
Male gender	-21.91214	14.32837	-1.53	0.369	-203.9713	160.147
**Number of RF lesions**						
Published year	0.3492074	0.1253937	2.78	0.219	-1.244071	1.942486
Research country	0.4072841	0.2026555	2.01	0.294	-2.167699	2.982267
Study design	1.166482	0.4745546	2.46	0.246	-4.863306	7.19627
Interventional parameter	-2.486559	1.047482	-2.37	0.254	-15.79608	10.82297
**Touch-up needed**						
Published year	0.0478391	0.0766701	0.62	0.645	-0.9263472	1.022025
Study design	-0.0552741	0.4386066	-0.13	0.920	-5.628299	5.517751
**Acute conduction recovery**						
Published year	0.0616289	0.6462428	0.1	0.939	-8.149665	8.272922
Research country	-0.3444556	3.792939	-0.09	0.942	-48.53832	47.84941
Study design	-0.1481234	1.674416	-0.09	0.944	-21.4236	21.12736
Interventional parameter	-10.58069	11.3521	-0.93	0.522	-154.8228	133.6614
Mean age	-4.791256	5.455508	-0.88	0.541	-74.11005	64.52754
Male gender	-9.694279	11.71157	-0.83	0.560	-158.5038	139.1153
**Recurrence rates**						
Published year	0.7603328	2.394938	0.32	0.804	-29.67024	31.1909
Research country	-0.3884593	1.125114	-0.35	0.788	-14.68438	13.90746
Study design	1.527826	4.029201	0.38	0.769	-49.66803	52.72368
Mean age	0.1156787	1.155708	0.1	0.936	-14.56898	14.80034
**Complications**						
Published year	-0.9955774	0.8425855	-1.18	0.447	-11.70164	9.710487
Research country	-0.7651117	0.8790133	-0.87	0.544	-11.93403	10.40381
Mean age	0.8118284	0.7133147	1.14	0.459	-8.251694	9.87535

Some covariates were not included in the meta-regression analysis of partial observational indicators because sensitivity analyses by eliminating studies one by one had been performed or the number of including studies was insufficient for analysis.

## 4. Discussion

In this study, we compared the efficacy and safety of CTI ablation between CF related parameters guidance and the conventional method. To our knowledge, it is the first meta-analysis on this issue. The main findings include the following: (1) ablation guided by CF related parameters significantly increases catheter tip-tissue contact; (2) it reduces RF duration and the number of RF lesions but does not significantly reduce total procedural time and fluoroscopy time; (3) it improves the success rate of bidirectional isthmus block after first ablation line achieved, but does not reduce the acute conduction recovery and recurrence rate; (4) it has the same safety compared with the conventional method.

Successful RF lesion formation has been shown to be dependent on transmural necrosis of target tissue, which is associated with several factors including temperature at the catheter-tissue interface, power and time of RF application, among them, good catheter-tissue contact is essential for the formation of effective lesion (9, 32, 33). Previous studies have shown that ablation guided by CF related parameters improved catheter-tissue contact in PVI, and reduced the acute gap formation and conduction recovery (34, 35), despite the latest meta-analysis has shown no significant reduction in recurrence rate (36). Although the value of CF related parameters has been confirmed in AF ablation, they cannot be considered to be identical fully in CTI ablation. Specifically, the structure of CTI is complicated, with the highly non-uniform thickness, as well as the presence of a prominent Eustachian ridge or sub-Eustachian pouches, which may alter the ability to create an adequate ablation lesion (31). Therefore, more stable contact is needed to achieve uniform and effective transmural lesions.

Previous studies have shown that the use of conventional parameters during CTI ablation in the absence of real-time CF sensing resulted in nearly half of all lesions being low CF with marked inhomogeneity of CF in different lesion regions (37). Low CF was implicated in more RF applications, longer time to achieve isthmus block, and increased risk of acute reconnection (27, 31). These findings underscored the importance of real-time CF measurements for optimizing ablation of typical AFL. As a comprehensive review of all relevant high-quality studies, this meta-analysis further confirmed that the use of CF related parameters offered CTI ablation more efficiency. Catheter-tissue contact was significantly increased when the CF was visible, thus reducing the time to acute isthmus block. At the same time, ablation guided by CF related parameters did not result in additional complications, or more steam pops. This is consistent with the results of previous studies of PVI and CTI ablation.

In this study, although no statistically significant differences were found between the two groups in terms of total procedural time and fluoroscopy time, there was a trend toward a decrease in all time parameters in the CF related parameters-guided ablation. We consider that it might be related to the small number of included studies and samples, i.e., sampling error leads to insignificant difference. Moreover, the inexperience of operators in CF related parameters-guided ablation could affect the operation efficiency, which might also lead to potential bias. However, based on the current evidence, CF related parameters guidance cannot be considered to shorten procedural time and fluoroscopy time in CTI ablation.

In terms of ablation effectiveness, although ablation guided by CF related parameters reduces the need for touch-up after completion of the first ablation line, acute reconnection and long-term outcomes are not improved. One possible reason for that is related to inappropriate CF. Specifically, although real-time CF is visible during RF application, the optimal CF value for CTI ablation still has not been confirmed, meaning that the contact may still be poor during ablation and cannot achieve transmural necrosis and lesion durability, thus gradual reconnection may occur after completion of the first ablation line. Furthermore, catheter stability and lesion continuity are also key elements influencing effective linear lesion formation (38, 39). That means excessively wide spacing between ablation points may result in incomplete electrical isolation, and catheter movement during RF application may result in insufficient ablation depth to reach transmural lesions.

In addition to maintain good catheter-tissue contact, adequate energy delivery causing thermal coagulation necrosis is also needed to achieve formation of a line of block across the CTI from the tricuspid annulus to the inferior vena cava, given the inhomogeneity of isthmus anatomy (40). Previous studies have shown that higher power can affect more effective isthmus lines by forming larger and deeper lesions, thus improve the long-term success rate of flutter ablation (41). But it is worth noting that higher power output is also potentially associated with a higher risk of complications, including coronary artery injury, pops, and even cardiac perforation (42). Moreover, the use of large-tip catheters has advantage of creating wider and deeper lesions than the conventional catheter (43). Thereby improving the continuity of lesions. The superior clinical efficacy of 8-mm tip electrode catheter compared to the conventional 4-mm catheter for flutter ablation has been confirmed by several studies (44). However, the optimal power setting has not been established for CF-guided and temperature controlled flutter ablation with large-tip catheters, and that should be investigated in the future to improve the overall efficacy of this procedure.

Recently, high-power short-duration (HPSD) ablation has been proposed as an effective and safe strategy for CTI ablation, allowing for more rapid superficial tissue ablation while avoiding complications associated with deeper lesions (45). Published studies showed the combined use of HPSD and CF technique was associated with a substantial reduction in total RF time compared to using only HPSD or traditional settings (46).

Notably, this meta-analysis involved three different contact parameters (CF, ECI, and AI), so we conducted a subgroup analysis to explore differences of their effects on CTI ablation. The result showed that effects of all three parameters were similar and did not significantly increase the inter-group heterogeneity, except for that on fluoroscopy time. Specifically, the only article involving AI showed a significant reduction in fluoroscopy time in the intervention group, while the other subgroups and the overall result were not statistically significant (29). We re-analyzed the methodology of this study and found that it was the only prospective, non-randomized study, as well as the only one involving Asians. Of note, the intracardiac echocardiography (ICE) was used in this study, which determined potential benefits in terms of reduction of ionizing radiation duration. The combination of CF related parameters and ICE may further improve ablation efficiency and reduce fluoroscopy time (47). More studies are needed in the future to confirmed it, although it does not have the crucial effect on the overall conclusion of this study.

Overall, CF related parameters offer possible incremental benefits in terms of efficiency without sacrificing safety and effectiveness. More studies are needed in the future to determine the exact optimal parameter values and verify its benefits of combination of it and other tools, as noted above.

## 5. Limitations

A limitation of this study is that some studies are of limited quality, given their characteristics such as non-RCTs, open-label design or funding from related instrument companies, that pose potential bias risk. Another limitation is that, moderate-high heterogeneity is found in some of the results, that should be interpreted with caution. It is associated with the quality of included data was not high enough. Although we have analyzed the source of heterogeneity, its effect on outcomes cannot be fully assessed, given differences of basic characteristics of patients and experience of operators. Furthermore, the limited data of included studies lead a lack of discussion about zero X-ray flutter ablation, a strategy with potential clinical benefits in terms of reduction of ionizing radiation exposure (47). With the rapid development of some facilitated tools, including 3D electroanatomic mapping systems, magnetic navigation and ICE, zero X-ray ablation should be more applied to improve safety of procedures and operators. Finally, due to the limited available ablation data, the effect of CF related parameters guidance on different anatomical segments of CTI during AFL ablation cannot be specifically analyzed.

## 6. Conclusion

CF related parameters guidance increases catheter-tissue contact in CTI ablation with the comparable safety as compared with conventional method, thus improves the effect of bidirectional isthmus block after first ablation line finished and reduces RF duration. However, it does not reduce the risks of acute conduction recovery and recurrent atrial arrhythmia.

## Data availability statement

The raw data supporting the conclusions of this article will be made available by the authors, without undue reservation.

## Author contributions

RW and NP: study concept and design. NP and JG: data search and extraction. NP, JG, and NZ: formal analysis and investigation. NP: writing – original draft preparation. NZ and MG: writing – review and editing. All authors contributed to the article and approved the submitted version.

## Supplementary material

Supplementary Figure 1 Forest plots for subgroup analysis of fluoroscopy time.
