# Supplementary figures and images for: Cavotricuspid isthmus ablation for atrial flutter guided by contact force related parameters: A systematic review and meta-analysis
Source: Front Cardiovasc Med. 2023 Jan 6;9:1060542. doi: 10.3389/fcvm.2022.1060542 (PMC9853203; doi:10.3389/fcvm.2022.1060542)

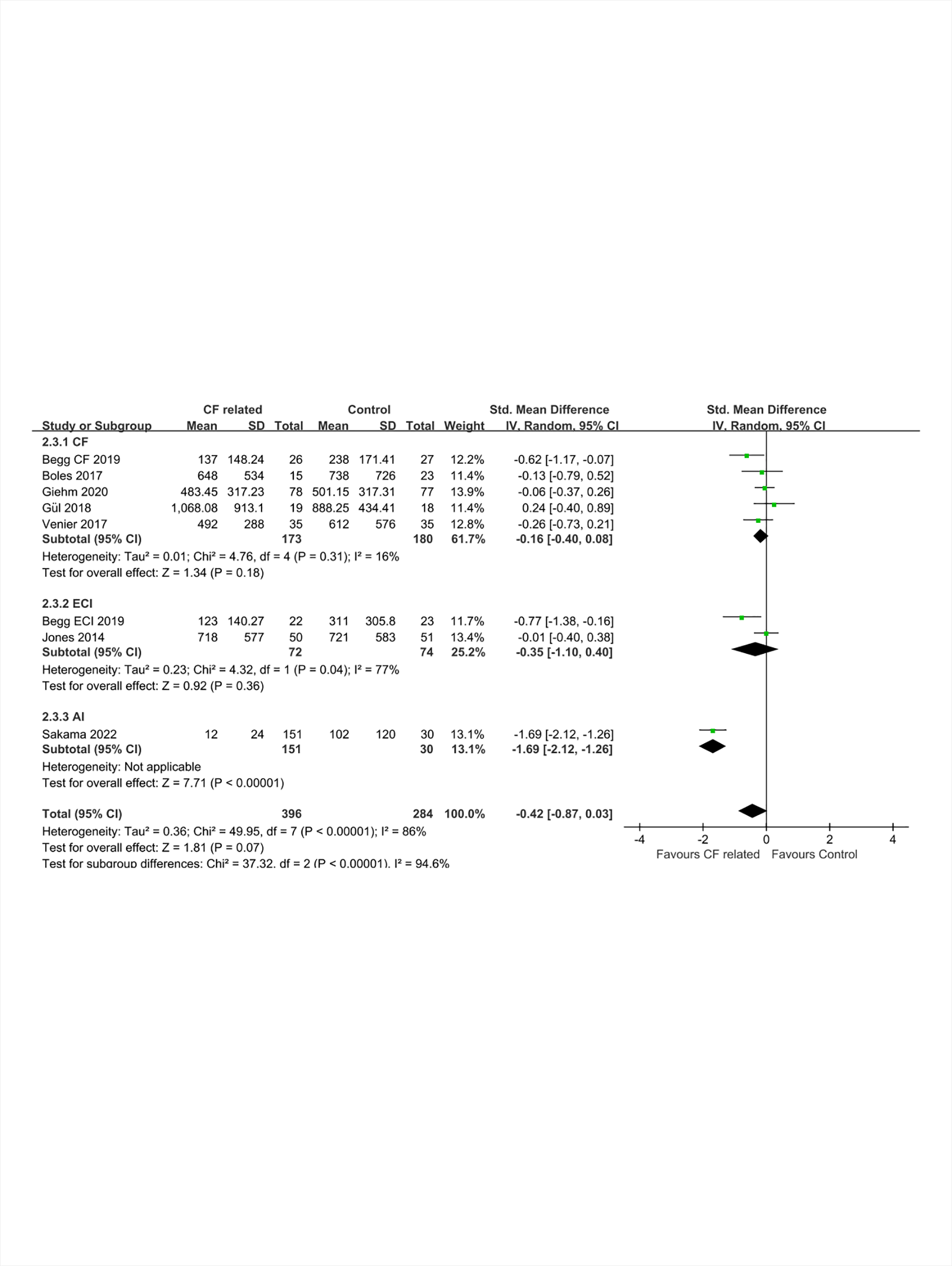

Supplement: Supplementary Figure 1 — Forest plots for subgroup analysis of fluoroscopy time. [file Image_1.TIF]
